# Age‐related increase in plasma p‐tau217 in amyloid‐beta–negative cognitively unimpaired individuals affects diagnostic interpretation

**DOI:** 10.1002/alz.71532

**Published:** 2026-06-06

**Authors:** Anna E Mammel, Fernando Gonzalez‐Ortiz, Ali Mousavi, Kelsey Hallett, Mary Encarnacion, Don Biehl, Pradip Gill, Sazan Ismael, Christopher Fowler, James D. Doecke, Larry Ward, Hans Frykman

**Affiliations:** ^1^ Neurocode USA Inc Bellingham Washington USA; ^2^ University of Gothenburg Gothenburg Sweden; ^3^ BC Neuroimmunology Laboratory Vancouver British Columbia Canada; ^4^ Division of Neurology Department of Medicine University of British Columbia Vancouver British Columbia Canada; ^5^ The Florey Institute of Neuroscience and Mental Health the University of Melbourne Parkville Victoria Australia; ^6^ The Australian e‐Health Research Centre Commonwealth Scientific and Industrial Research Organisation Brisbane Queensland Australia; ^7^ School of Medical and Health Sciences Edith Cowan University Joondalup Western Australia Australia; ^8^ National Reference Lab Abu Dhabi UAE

**Keywords:** Alzheimer's disease, biomarkers, cognitively normal, normal aging, plasma p‐tau217

## Abstract

**INTRODUCTION:**

The extent to which non‐pathological aging influences plasma biomarkers remains unclear. Here, we investigate factors influencing plasma p‐tau217 levels in cognitively unimpaired (CU), amyloid‐beta–negative (Aβ‐) individuals.

**METHODS:**

Plasma p‐tau217 was measured in CU Aβ‐ positron emission tomography–negative (PET‐) participants using two immunoassays (ALZpath *n* = 360 and LUMIPULSE G1200 *n* = 73). Associations between p‐tau217 and age groups (60–69, 70–79, and 80+ years), apolipoprotein E (*APOE)* genotype, and gender were evaluated.

**RESULTS:**

ALZpath plasma p‐tau217 showed a non‐pathological age‐related increase (*p* < 0.001), and increased percentage within the intermediate zone with age. Lumipulse p‐tau217 showed an increase in the percentage of subjects with positive results with age, however, this trend was not significant (*p* > 0.05). Men had higher levels of p‐tau217 only with the ALZpath assay (*p* = 0.02; Lumipulse: *p* = 0.81). No significant associations were found between p‐tau217 and *APOE* genotype.

**DISCUSSION:**

Our results highlight the importance of incorporating age and sex into the interpretation of plasma p‐tau217 particularly in preclinical stages.

## Highlights

- Plasma p‐tau217 increases with non‐pathological aging in cognitively unimpaired (CU) amyloid‐beta–negative (Aβ‐) individuals.
- ALZpath p‐tau217 intermediate‐zone rates increase with age.
- Lumipulse p‐tau217 shows age‐related positivity trends without significance.
- Sex differences observed with ALZpath p‐tau217; no apolipoprotein E (APOE) associations.

## 1 BACKGROUND

Emerging advancements in blood‐based biomarkers to detect abnormal amyloid levels associated with Alzheimer's disease (AD) provide affordable, less‐invasive and scalable alternatives to positron emission tomography (PET) or cerebrospinal fluid (CSF) biomarker measurements. ^1^, ^2^, ^3^ Notably, plasma p‐tau217 has become a core biomarker to aid in the diagnosis of AD due to its strong correlations with hallmark AD pathologies determined either by CSF biomarker abnormalities, PET, or post‐mortem examination,^4^ and has also been shown to differentiate AD from other forms of dementia and tauopathies.^5^, ^6^, ^7^, ^8^ Furthermore, it has also shown high accuracy in identifying AD pathology in cognitively unimpaired (CU) individuals.^9^, ^10^ The extensive validation performed by our laboratory, and other research groups across a range of cohorts, has led to plasma p‐tau217 to be considered a core 1 biomarker in the Alzheimer's Association revised criteria.^8^, ^11^, ^12^, ^13^ Understanding the baseline level of p‐tau217 in CU individuals and establishing a clinical reference range is critical, as clinical trials are increasingly using plasma p‐tau217 for recruitment and evaluating early drug intervention.^14^, ^15^ However, our understanding of the mechanisms affecting the concentrations of plasma p‐tau217 concentrations in older‐aged CU individuals remains limited.^16^

In addition, AD biomarkers are known to be influenced by non‐modifiable risk factors of AD including age, sex, ethnicity, apolipoprotein E epsilon 4 (*APOE* ε4) and other covariates such as renal disease, cardiovascular disease, body mass index and hypertension.^17^ Therefore, to confidently interpret plasma p‐tau217 in the biological diagnosis of AD, particularly in its preclinical stage, we need to understand how AD risk factors are associated with plasma p‐tau217 in the general population. In this study, we investigated the impacts of age, sex, *APOE* ε4 genotypes, and particularly age‐related changes in plasma p‐tau217, among a large‐ scale cohort of amyloid‐beta–negative (Aβ−) CU elderly people and examine how aging influences p‐tau217 positivity rates within this population.

## 2 METHODS

### 2.1 Participants

Participants were recruited from the Australian Imaging, Biomarker & Lifestyle study of ageing (AIBL). Since 2006, AIBL has collected data from 3045 participants with 10,494 person‐contact years. The study collects information from the cohort every 18 months on biomarkers from blood and CSF (when available), as well as PET and magnetic resonance imaging (MRI) imaging. AIBL researchers also collect information on cognition, exercise, diet, and health and lifestyle factors that may contribute to the development of AD.^18^ Amyloid status was determined by Aβ‐PET, all subjects included in this study had Centiloid (CL) < 9.9 and classified by negative (< 15 CL).

### 2.2 Plasma sampling and plasma p‐tau217 measurement

Plasma samples collected and stored according to AIBL protocol^18^ were obtained and plasma p‐tau217 was measured using ALZpath Simoa p‐tau217 v2 (Quanterix, MA, USA) on the Quanterix HD‐X Analyzer platform in duplicate. Three internal quality controls were used, and all values were within ± 15% of the established ranges.^19^ Plasma samples were also measured using Lumipulse G pTau217 Plasma (Fujirebio, PA, USA) on the LUMIPULSE G1200 platform in singlicate. Two manufacturer‐provided controls were used, and all control values were within the manufacturer‐provided ranges.^19^

### 2.3 Statistical analysis

For descriptive analysis, categorical variables were summarized using percentages, and interval variables were reported as mean ± standard deviation (SD). Comparison based on sex were performed using Welch's two‐sample *t*‐test. For interval variables, we used one‐way analysis of variance (ANOVA), followed by the Tukey's honestly significant difference (HSD) post hoc test for comparing group means. To evaluate associations between plasma biomarker concentrations and demographic or clinical variables, the p‐tau217 values were log‐transformed to improve normality and stabilize variance. The resulting plasma p‐tau217 concentration dependent variable was modeled using a generalized linear model (GLM) with the following predictors: age, sex, *APOE* ε4 carrier status, years of education, Mini‐Mental State Examination (MMSE) score, and Clinical Dementia Rating Global (CDR‐G) score. Outliers were assessed in the context of the regression model using studentized residuals and the Bonferroni‐adjusted p‐value. Two subjects were identified as statistically significant outliers (Bonferroni‐adjusted *p* < 0.05). To determine the influence of these outliers on the results, models were re‐run excluding the outlying data points. Only the model for MMSE was affected by outlier removal, however no significant difference was seen in model estimates from either inclusion or removal of the two outliers. Dual cutoff values for the ALZpath p‐tau217 to determine the proportions of participants in the intermediate zone were taken from Mammel et al, (2025). Given the smaller sample size for the Lumipulse assay, participants were not assessed using the dual cutoff approach. Statistical analyses were completed using SPSS 29 and R (version 4.3.1).

**RESEARCH IN CONTEXT**

1. **Systematic review**: Plasma p‐tau217 has been established across multiple cohorts as a core biomarker for amyloid pathology. However, few studies have examined p‐tau217 levels in cognitively unimpaired, amyloid‐negative older adults. Limited evidence exists regarding how age, sex, apolipoprotein E (APOE) ε4, and other risk factors influence p‐tau217 concentrations in this population.
2. **Interpretation**: Our findings demonstrate that plasma p‐tau217 shows age‐related increases even in the absence of amyloid pathology, with assay‐specific differences in sex effects and no influence of APOE genotype. These results refine the understanding of non‐pathological contributors to p‐tau217 variability and highlight the need to consider demographic factors when interpreting biomarker results in preclinical populations.
3. **Future directions**: Future work should determine the biological mechanisms underlying age‐related p‐tau217 increases and evaluate longitudinal trajectories across assays. Establishing age‐ and sex‐adjusted thresholds may improve biomarker interpretation in early disease stages.

## 3 RESULTS

### 3.1 Clinical characteristics of study participants

AIBL participant demographics are presented in Table 1. Of the 360 participants included in this study, the majority (*n* = 201, 55.8%) were female. Participants were on average 72.5 (± 5.2) years of age, (female: 71.6 ± 5, male: 73.8 ± 5). All participants had normal cognitive function with an average of MMSE 28.9 (± 1.1) and a CDR‐G score of 0.01 (± 0.08; 9/360 had CDR‐G 0.5). Average years of education was 13.2 (± 3.1) years (females 13.7 [± 3.1], males 12.8 [± 3]). Among detected *APOE* genotypes, the most common genotype was ε3/ε3 (*n* = 238, 66.1%); 20.3% of participants (*n* = 73) were carriers for the *APOE* ε4 allele, and the penetrance for the *APOE* ε4 allele was similar between males and females (*p* = 0.6). In addition, there was no significant association between sex and *APOE* ε4 carrier status.

**TABLE 1:** Demographics of cognitively unimpaired cohort.

Demographics	Mean ± SD
Age	72.5 ± 5.2 years
Sex, female no. (%)	201 (55.8%)
APOE e4 carriers, no. (%)^a^	73 (20.3%)
Years of education	13.2 ± 3.1 years
MMSE	28.9 ± 1.1
CDR score	0.01 ± 0.08
Amyloid PET negative, no. (%)	360 (100%)
ALZpath pTau217 conc.	0.24 ± 0.14 pg/mL
Lumipulse pTau217 conc.^b^	0.10 ± 0.05 pg/mL

Abbreviations: APOE, apolipoprotein E; CDR, Clinical Dementia Rating; MMSE, Mini‐Mental State Examination; PET, positron emission tomography; SD, standard deviation.

^a^APOE genotyping available for 341 subjects.

^b^Testing results for 73 subjects.

### 3.2 Effect of patient demographics on p‐tau217 levels in CU Aβ‐ individuals

**FIGURE 1:**
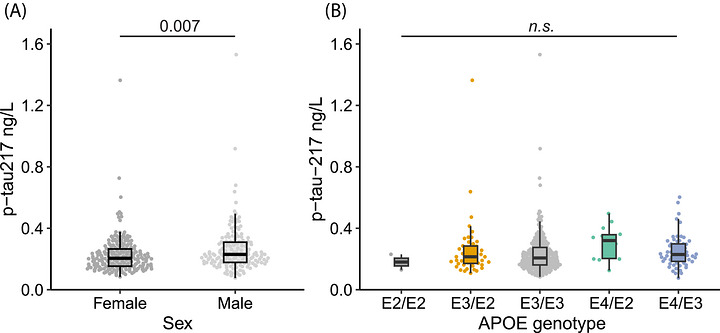
Cognitively unimpaired amyloid‐negative demographics and plasma p‐tau217 concentrations. (A) ALZpath p‐tau217 levels based on sex; Welch's *t*‐test; *p*‐value = 0.007; *N* = 201 females and *N* = 159 males. (B) ALZpath p‐tau217 concentration by APOE genotypes; ANOVA; *p*‐value > 0.05; *N* = 2 ε2/ ε2, *N* = 47 ε3/ ε2, *N* = 238 ε3/ ε3, *N* = 14 ε4/ ε2, and *N* = 59 ε4/ ε3. ANOVA, analysis of variance; APOE, apolipoprotein E.

We determine whether patient demographics including sex, *APOE* genotype, and cognitive score impacts p‐tau217 concentration in Aβ negative individuals. We observed a difference in the ALZpath plasma p‐tau217 contraction based on sex, with males having slightly higher p‐tau217 levels compared with females (*p* = 0.007) (Figure 1A), however the same was not seen for the Lumipulse assay (p = 0.85) (Figure S1A). We next examined if p‐tau217 levels were affected by *APOE* genotype. There was no difference in p‐tau217 concentration between ε2/ε2, ε2/ε3, ε3/ε3, ε4/ε3 genotypes for both ALZpath and Lumipulse (ANOVA; *p*‐value > 0.05), suggesting in CU Aβ‐ individuals, *APOE* genotype does not affect p‐tau217 levels (Figure 1B and Figure S1B). Assessment of ALZpath pTau217 levels with MMSE saw no significant changes with decreasing levels of MMSE (*p* = 0.17), however there was a weak but significant increase in Lumipulse pTau217 levels with increasing MMSE (*p* = 0.02) (data not shown). All other demographic effects were not impacted by outlier removal.

### 3.3 Age‐associated increase in p‐tau217 levels in CU Aβ‐ individuals

**FIGURE 2:**
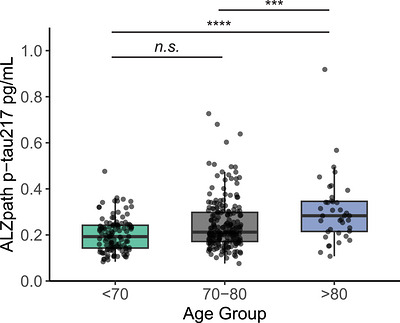
Plasma p‐tau217 concentration stratified by age group in CU Aβ‐ subjects ALZpath p‐tau217 concentration stratified by age group; ANOVA; *p*‐value < 0.0001; *N* = 115 age < 70, *N* = 205 age 70–80, and *N* = 40 age > 80 years old. *N* = 2 outlier data points not shown. ANOVA, analysis of variance; CU, cognitively unimpaired.

The effect of age on p‐tau217 levels for Aβ‐ CU individuals was determined by partitioning the subjects into three age groups: < 70, 70–80, > 80 years of age. We observed that plasma p‐tau217 levels (ALZpath) increased with age from a median concentration of 0.19 ng/L with a reference range of 0.10–0.36 ng/L for subject < 70 years, to a median of 0.34 ng/L with a reference range between 0.12–0.93 ng/L for individuals > 80 years old (Table 2). The reference range was defined as the 2.5th to 97.5th percentile. There was a significant increase in p‐tau217 concentrations for individuals aged > 80 years compared to individuals aged < 70 years (*p*‐value < 0.0001) and aged 70‐80 (*p* < 0.004) (Figure 2). There was also a significant increase in p‐tau217 values between < 70 age and 70–80 age groups (*p* = 0.0004), suggesting a stepwise increase in pTau217 levels with increasing age.

**TABLE 2:** ALZpath p‐tau217 performance as stratified by age.

	Age groups (years)			
Parameter	<70	70–80	>80	All
Median	0.19	0.21	0.28	0.21
Reference interval (2.5–97.5th)	0.10–0.36	0.11–0.51	0.12–0.93	0.11–0.51
Negative (≤0.34 ng/L)	93.0%	85.4%	62.5%	85.3%
Intermediate (0.34–0.63 ng/L)	6.1%	13.2%	32.5%	13.1%
Positive (>0.63 ng/L)	0.9%	1.5%	5.0%	1.7%
Total *N*	115	205	40	360

To determine if the age dependent effects are assay specific we tested a subset of subjects with sufficient sample volume using the Fujirebio Lumipulse p‐tau217 assay. The same age dependent effects observed with the ALZpath p‐tau217 were not observed with the Lumipulse assay, however, these results require further investigation as the number of samples analyzed in the Lumipulse analysis was lower (Figure S2).

### 3.4 Increases in indeterminate p‐tau217 results in CU Aβ‐ individuals with age

We evaluated whether the age‐dependent increase in p‐tau217 impacts the proportion of participants in each of the negative, intermediate, and positive zones for each age group. Cutoffs were defined previously in Mammel et al. 2025 where results < 0.34 ng/L were negative, between 0.35 and 0.62 ng/L were defined as intermediate, and results ≥ 0.63 ng/L were positive. For individuals age 60–69, 92.4% were negative, for age 70–79, 85.4% were negative, and lastly for those older than 80 years of age, 62.5% were negative (Table 2). The positive rate remained at 5% or below across all age groups, and the risk of false‐positives remains low despite the age dependent‐increase by using a two‐cutoff approach.

Most notable was the increase in the proportion of participants in the intermediate zone. This increased for each age group but remained below the 20% threshold set by CEOi for all age groups < 80 years.^20^ The proportion in the intermediate zone exceeded current recommendations, with 32.5% of individuals age 80–85 falling into this zone (Table 2). The overall negative rate was 85.3%, with 13.1% in the intermediate zone, and 1.7% positive across the entire sample population (Table 2).

For the Lumipulse assay, the percentage of participants who had a positive test result (pTau217 > 0.18 ng/mL) increased from 0% for individuals at < 70 years, 4.7% for individuals age 70–80, and 11.1% for > 80 years. The overall positivity rate was 4.1% for the Lumipulse p‐tau217 in this cohort (Table S1).

## 4 DISCUSSION

According to the 2024 revised criteria from the Alzheimer's Association, plasma p‐tau is considered a core 1 biomarker, meaning that elevations in plasma p‐tau181, p‐tau217, or p‐tau231 are sufficient to establish a biological diagnosis of AD.^11^ In this study, we assessed plasma p‐tau217 levels in CU Aβ‐ older adults to investigate how non‐pathological aging and AD risk factors may influence p‐tau217 levels. We found a clear age‐related increase in the ALZpath plasma p‐tau217 concentrations among amyloid‐negative CU individuals, particularly pronounced in those over 80 years of age. This increase was independent of sex or *APOE*‐ε4 genotype. Results from the present study focused upon the assessment of the ALZpath p‐tau217 data, with a small replication using the Lumipulse p‐tau217 assay. Given the small sample size of the replication, we recognize the limitations of concluding substantial evidence from this work. Thus, assessment of results from the Lumipulse data is presented here as descriptive evidence only, with small to moderate, yet non‐significant increases seen in p‐tau217 levels between the participants younger than 70, compared with those aged 70 or older. However, age dependent increases have been observed for the Lumipulse p‐tau217 and p‐tau217/Ab42 ratio.^21^ This suggests that the effects of aging may be universal and not platform specific, and may have potential impacts on current United States Food and Drug Administration (FDA) ‐approved diagnostic tests. If age‐related increases are not considered, older individuals may be more likely to yield intermediate or borderline positive results despite lacking objective evidence of amyloid deposition. This could complicate the diagnosis for clinicians for patients over age 80 and result in costly and potentially unnecessary follow‐up testing for these individuals.

While plasma p‐tau217 has been widely validated as a promising biomarker for AD, our results indicate that age‐related increases can occur even in the absence of amyloid pathology, supporting previous research showing that small elevations in plasma p‐tau217 are not always indicative of AD.^22^, ^23^, ^24^, ^25^, ^26^, ^27^ Moreover, recent evidence has shown that, even among Aβ+ individuals, plasma p‐tau217 positivity does not necessarily predict clinical progression as a standalone test without repeat measurement.^28^ Lastly, age‐dependent increases in p‐tau217 have been shown in previous studies; however, these were attributed to Aβ deposition.^29^, ^30^ Our results demonstrate that this increase is occurring independent of Aβ, and could be related to an age‐dependent increase in peripheral tau production that may not be related to AD disease pathology, future studies will be needed to determine if brain and/or peripheral tau specific isoforms increase with age.

These findings have important implications for the use of current p‐tau217 assay as diagnostic tools in clinical practice and as a screening or enrichment in AD clinical trials, especially when relying on universal cutoffs. Our findings highlight caution when interpreting plasma p‐tau217 alone without proper clinical context, particularly in older populations. Sex and age‐dependent effects are currently lacking in the Alzheimer's Association criteria.^11^ Developing dynamic cutoffs or incorporating complementary biomarkers (e.g., Aβ42/40 ratios, glial fibrillary acidic protein [GFAP], neurofilament light chain [NfL]) or CSF and PET data could improve diagnostic specificity and the effect of age‐related changes.^12^, ^31^

Altogether, our results underscore the importance of interpreting plasma p‐tau217 levels within the appropriate clinical context, with careful consideration of age as a potential driver of non‐pathological increases. Future studies should aim to replicate these findings in larger and more diverse populations and conduct longitudinal analyses to determine the clinical significance of elevated p‐tau217 in aging individuals without evidence of amyloid pathology.

## CONFLICT OF INTEREST STATEMENT

Anna E Mammel, Kelsey Hallett, Don Biehl, Pradip Gill, and Fernando Gonzalez‐Ortiz are employees of Neurocode USA, Inc. Neurocode was involved in the writing and editorial support of this article. Hans Frykman is a full owner of BC Neuroimmunology (BCNI) and a partial owner of Neurocode USA, Inc. Mary Encarnacion and Ali Mousavi are employees of BCNI. Sazan Ismael, Christopher Fowler, James D. Doecke, and Larry Ward have no conflicts of interest. Author disclosures are available in the supporting information

## CONSENT STATEMENT

All participants completed informed consent after discussing the study in detail with a senior member of the research team and 80 mL of blood was drawn in the mornings.^18^ The AIBL study was approved by the institutional ethics committees of Austin Health, St Vincent's Health, Hollywood Private Hospital and Edith Cowan University, and all volunteers gave written informed consent before participating in the study.^18^

## Disclosure

Author disclosures are available in the supporting information.

## Supporting information




Supporting Information

Supporting Information
