# Supplementary material for: Age‐related increase in plasma p‐tau217 in amyloid‐beta–negative cognitively unimpaired individuals affects diagnostic interpretation
Source: Alzheimers Dement. 2026 Jun 6;22(6):e71532. doi: 10.1002/alz.71532 (PMC13242609; doi:10.1002/alz.71532)

**Supplemental Data**


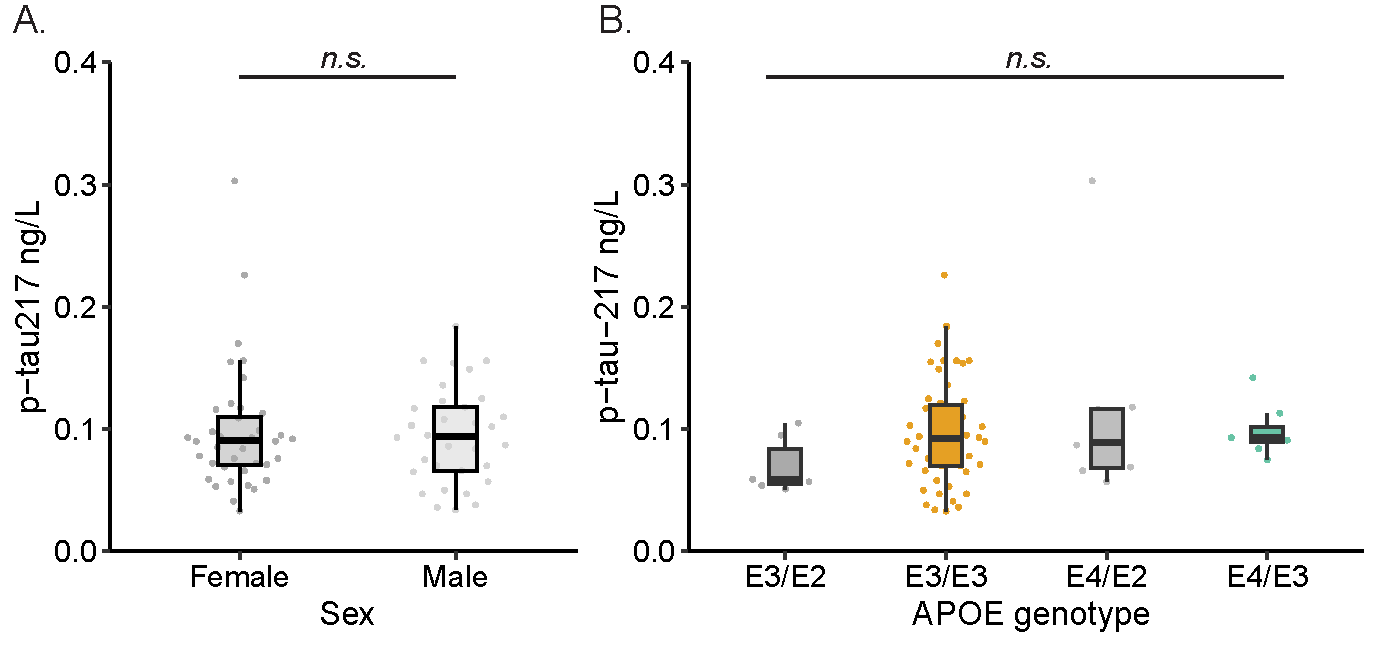
Supplemental Figure 1: Lumipulse p-tau217 concentrations by sex and APOE genotype CU Aβ- subjects. (A) Lumipulse p-tau217 levels based on sex, Welch’s t-test; p-value > 0.05; N = 40 females and N = 33 males. (B) Lumipulse p-tau217 levels by ApoE genotype; ANOVA; p-value > 0.05; N = 0 ε2/ ε2, N = 7 ε3/ ε2, N = 50 ε3/ ε3, N = 8 ε4/ ε2, and N = 8 ε4/ ε3.


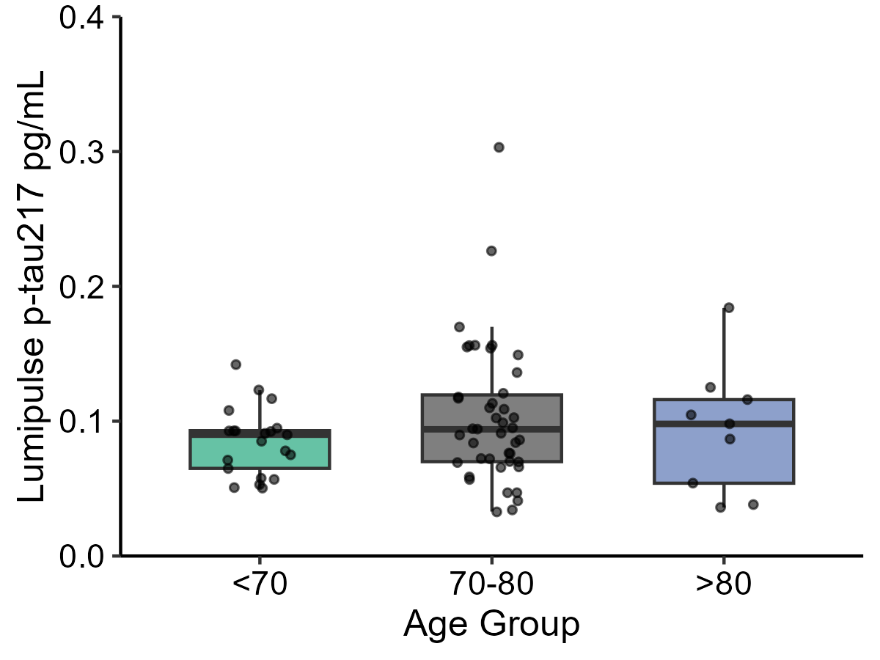


Supplemental Figure 2: Lumipulse p-tau217 concentration stratified by age group; ANOVA; p-value = 0.45; N = 21 age 60 – 69, N = 43 age 70 – 79, and N = 9 age > 80 years old.


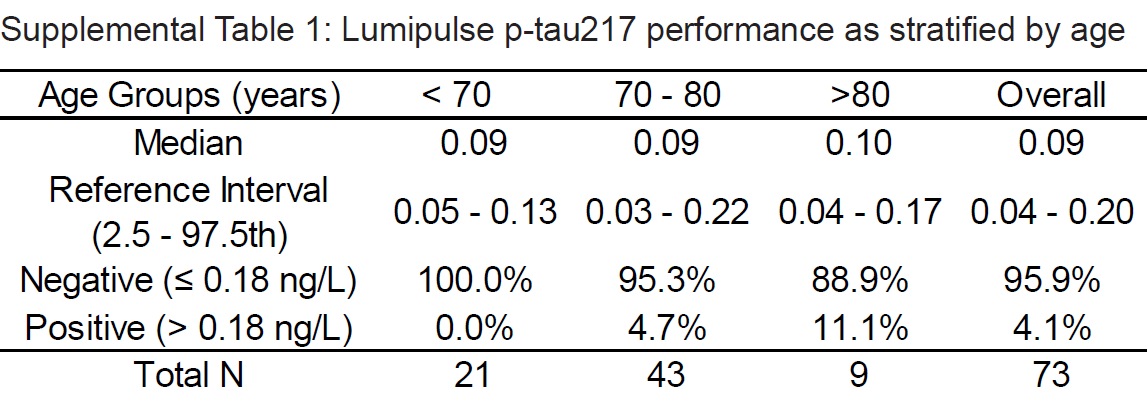

Supplement: Supplementary file 1 — Supporting Information [file ALZ-22-e71532-s002.docx]
